# Co-Morbid Insomnia and Sleep Apnea (COMISA): Prevalence, Consequences, Methodological Considerations, and Recent Randomized Controlled Trials

**DOI:** 10.3390/brainsci9120371

**Published:** 2019-12-12

**Authors:** Alexander Sweetman, Leon Lack, Célyne Bastien

**Affiliations:** 1 The Adelaide Institute for Sleep Health: A Flinders Centre of Research Excellence, Box 6 Mark Oliphant Building, 5 Laffer Drive, Bedford Park, Flinders University, Adelaide 5042, South Australia, Australia; 2 The Adelaide Institute for Sleep Health: A Flinders Centre of Research Excellence, College of Education Psychology and Social Work, Flinders University, Adelaide 5042, South Australia, Australia; leon.lack@flinders.edu.au; 3 School of Psychology, Félix-Antoine-Savard Pavilion, 2325, rue des Bibliothèques, local 1012, Laval University, Quebec City, QC G1V 0A6, Canada; celyne.bastien@psy.ulaval.ca

**Keywords:** COMISA, insomnia, obstructive sleep apnea, sleep-disordered breathing, cognitive behavior therapy for insomnia, continuous positive airway pressure

## Abstract

Co-morbid insomnia and sleep apnea (COMISA) is a highly prevalent and debilitating disorder, which results in additive impairments to patients’ sleep, daytime functioning, and quality of life, and complex diagnostic and treatment decisions for clinicians. Although the presence of COMISA was first recognized by Christian Guilleminault and colleagues in 1973, it received very little research attention for almost three decades, until the publication of two articles in 1999 and 2001 which collectively reported a 30%–50% co-morbid prevalence rate, and re-ignited research interest in the field. Since 1999, there has been an exponential increase in research documenting the high prevalence, common characteristics, treatment complexities, and bi-directional relationships of COMISA. Recent trials indicate that co-morbid insomnia symptoms may be treated with cognitive and behavioral therapy for insomnia, to increase acceptance and use of continuous positive airway pressure therapy. Hence, the treatment of COMISA appears to require nuanced diagnostic considerations, and multi-faceted treatment approaches provided by multi-disciplinary teams of psychologists and physicians. In this narrative review, we present a brief overview of the history of COMISA research, describe the importance of measuring and managing insomnia symptoms in the presence of sleep apnea, discuss important methodological and diagnostic considerations for COMISA, and review several recent randomized controlled trials investigating the combination of CBTi and CPAP therapy. We aim to provide clinicians with pragmatic suggestions and tools to identify, and manage this prevalent COMISA disorder in clinical settings, and discuss future avenues of research to progress the field.

## 1. Introduction

### 1.1. Insomnia and Obstructive Sleep Apnea

Insomnia and obstructive sleep apnea (OSA) are the two most common sleep disorders, which both include nocturnal sleep disturbances, impairments to daytime functioning, mood, and quality of life, and high healthcare utilization [1]. As this review focuses on patients with co-morbid insomnia and sleep apnea (COMISA), a brief introduction to both insomnia and OSA is provided below.

Insomnia disorder is characterized by frequent and chronic self-reported difficulties initiating sleep, maintaining sleep, and early morning awakenings from sleep, which are associated with impaired daytime functioning, mood, and quality of life [1,2]. Insomnia disorder is thought to result from a combination of pre-disposing, precipitating, and perpetuating factors, and is conceptualized as a self-perpetuating disorder including elevated cognitive and physiological ‘hyper-arousal’ [3,4,5]. The estimated prevalence of insomnia varies widely according to diagnostic criteria and specific populations of interest, however it is thought that 6%–10% of the general population suffer from chronic insomnia disorder, which includes clinically significant and frequent nocturnal sleep disturbances and impaired daytime functioning [6,7]. Although cognitive and behavioral therapy for insomnia (CBTi) leads to long-term improvement of insomnia and is the recommended ‘first line’ insomnia treatment [8,9,10,11], a lack of access to CBTi has resulted in the majority of insomnia sufferers receiving prescriptions for sedative-hypnotic medications as the initial and ongoing treatment [12,13].

Alternatively, OSA is characterized by repetitive brief closure (apnea) or narrowing (hypopnea) of the pharyngeal airway during sleep which result in the cessation or reduction of airflow, reduced oxygen saturation, and commonly terminate in post-apneic arousals from sleep, increased sympathetic activity, and the resumption of airflow [1,14,15]. OSA is thought to result from a combination of anatomical (e.g., a narrow pharyngeal airway), and non-anatomical factors (e.g., impaired upper airway muscle function, low arousal threshold, and unstable control of breathing) [16]. The combination of frequent respiratory events and arousals from sleep throughout the night severely fragments sleep architecture, resulting in perceptions of chronically non-restorative sleep, reduced quality of life, excessive daytime sleepiness, and increased risk of motor-vehicle accidents [17,18,19]. The most common index of OSA presence and severity is the apnea/hypopnea index (AHI), which represents the average number of respiratory events experienced per hour of sleep. Diagnostic criteria for OSA include an AHI of at least five in the presence of an associated complaint/disorder (e.g., insomnia, sleepiness, fatigue, snoring, hypertension, atrial fibrillation, congestive heart failure, etc.), or an AHI of at least 15 [1]. Mild, moderate, and severe OSA are diagnosed according to an AHI of ≥5 to <15, ≥15 to <30, and ≥30, respectively [15]. The prevalence of OSA varies by diagnostic criteria and the sample population, however it is estimated that approximately 10% of the general population fulfil diagnostic criteria for OSA [20]. The most effective treatment for moderate and severe OSA is continuous positive airway pressure (CPAP) therapy, which stabilizes breathing throughout the night, improves daytime sleepiness and quality of life, and reduces risk of motor-vehicle accidents [15,19,21,22]. However, CPAP therapy requires patients to wear pressurized nasal/oro-nasal masks throughout the night, and is limited by poor patient acceptance and disappointing long-term adherence [23].

### 1.2. The Beginning of COMISA Research

**Figure 1:**
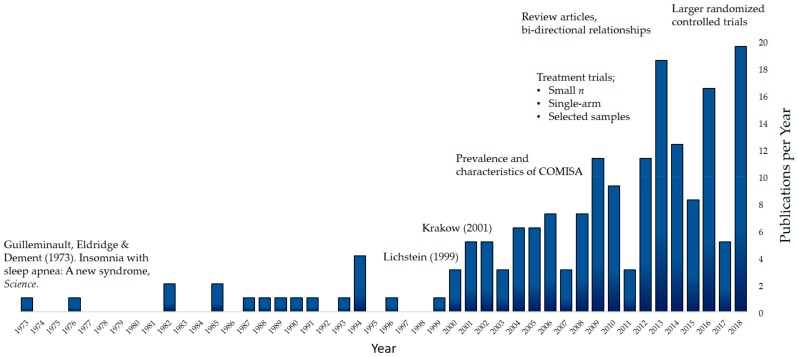
History of research in co-morbid insomnia and sleep apnea, including Guilleminault and colleague’s 1973 article, and a lack of widespread research attention until two articles by Lichstein and colleagues (1999) and Krakow and colleagues (2001).

Guilleminault, Eldridge, and Dement were the first to document the co-occurrence of insomnia and sleep apnea in 1973 [24]. Two middle-aged male patients were described, who presented with histories of chronic sleep maintenance and early morning awakening insomnia complaints. Both patients completed overnight polysomnography (PSG) studies at the Stanford Sleep Disorders Clinic, and were subsequently found to have significant sleep apnea. In 1976, Guilleminault and colleagues conducted a larger study to identify the proportion of chronic insomnia patients with occult OSA [25], and reported that of the 56 patients referred for symptoms of chronic insomnia, 10.7% were also found to have sleep apnea. Given that many insomnia patients are prescribed sedating benzodiazepine medications that can potentially exacerbate manifestations of OSA, Guilleminault and colleagues expressed substantial concern regarding the identification and management of COMISA patients [25]. Hence, this early COMISA research highlighted the importance of assessing insomnia patients for OSA symptoms, and recommended additional research to investigate the overlap of insomnia and OSA [24,25]. However, this prescient suggestion failed to attract research interest in the COMISA field for the subsequent three decades (Figure 1). 

This lack of research attention possibly resulted from the differences in stereotypical characterizations of OSA and insomnia patient profiles, which mis-directed clinical attention, and research interest in their co-occurrence. For example, OSA has historically been conceptualized as a disorder impacting middle-aged and older-adult males, who are overweight or obese and present with complaints of excessive daytime sleepiness, tiredness, sedation, and snoring. Although epidemiological studies provide statistical support for these ‘risk factors’ (e.g., OSA is associated with male gender, increased age, overweight and obesity, snoring, and daytime sleepiness [14]), reliance on this single profile clearly does not represent all OSA sufferers [26,27,28]. Alternatively, insomnia has historically been conceptualized as a disorder primarily impacting middle-aged and older-adult females who are predisposed to anxiety, stress, and cycles of cognitive rumination. Although cross-sectional studies support the statistical association of these age, gender and personality characteristics with insomnia symptoms, this profile is not reflective of all insomnia sufferers [6]. Given that several risk factors and symptoms of OSA and insomnia occur in direct opposition (e.g., male vs. female sex, sleepiness vs. sleeplessness, sedation vs. anxiety), it seems counterintuitive that the two disorders should co-occur. Consequently, these historical conceptualizations of distinct insomnia- and OSA-profiles may have contributed to an attentional and referral bias against COMISA, which resulted in the 27 year period of research dormancy.

Alternatively, the lack of COMISA research following Guilleminault’s 1973 publication may have also resulted from conceptualizations of ‘primary’ versus ‘secondary’ insomnia. Historically, when insomnia has co-occurred in the presence of another disorder, it has commonly been conceptualized as a ‘secondary’ condition, which is precipitated and maintained by the assumed ‘primary’ disorder [29]. This assumption implies that insomnia complaints will improve with successful treatment of the assumed ‘primary’ condition, and that the insomnia does not require independent diagnostic attention or targeted treatment [29,30,31,32]. Although insomnia is now recognized as an independent and self-perpetuating disorder which necessitates targeted diagnostic and therapeutic attention in the presence of co-occurring disorders [32,33], these historical mis-conceptualizations of ‘secondary’ insomnia may have also reduced research interest in COMISA during this period.

Although a handful of articles reported on the co-occurrence of insomnia and OSA following Guilleminault’s seminal 1973 publication [34,35,36], widespread interest in COMISA was only re-ignited following two articles published in 1999 and 2001. In 1999, Lichstein and colleagues examined the incidence of OSA among 80 insomnia patients without obvious indicators of OSA (i.e., patients with symptoms indicative of OSA, including witnessed apneas, snoring, excessive daytime sleepiness, obesity, etc., were excluded from the study) [37]. Of the 80 insomnia patients, 43% had an AHI of at least five (indicative of at least mild OSA), and 29% had an AHI of at least 15 (indicative of at least moderate OSA) on an overnight PSG study. Alternatively, in 2001 Krakow and colleagues reported that among 231 OSA patients managed at a University Sleep Clinic 50% reported clinically significant insomnia symptoms, including at least two symptoms of either taking at least 30 min to fall asleep, waking up a lot, or having difficulty returning to sleep upon awakening [38]. Collectively, these two articles provided substantial evidence of the common co-morbidity of insomnia and OSA first reported by Guilleminault in 1973 [24], and provided a springboard for additional COMISA research. Figure 1 provides an overview of primary research, review articles, and conference abstracts in the COMISA research field per year, since Guilleminault’s initial *Science* publication. In this narrative review, we plan to update and extend upon two previous reviews in the COMISA field [39,40], to describe the most recent and relevant research investigating the prevalence, consequences, methodological considerations, treatment approaches, and theoretical bi-directional relationships in COMISA. This recent research is integrated with previous literature to provide researchers and clinicians with clinical recommendations and future research directions. 

### 1.3. COMISA Prevalence

The frequent co-occurrence of insomnia and OSA has since been confirmed by many research groups who have investigated a wide variety of samples and utilized a number of different tools and criteria to define insomnia, and OSA. In 2010 Luyster and colleagues reviewed COMISA literature, and concluded that 39%–58% of OSA patients report insomnia symptoms, and 29%–67% of insomnia patients fulfil minimal criteria for OSA [39]. In 2017, we subsequently published a review article in which we coined the term “COMISA”, and reviewed prevalence estimates reported from 2010 to 2015, consequences, and early COMISA treatment research [40]. In our review of prevalence studies, we confirmed the high prevalence of COMISA, which varies considerably based on the initial disorder of interest (e.g., the prevalence of OSA in insomnia patients, or the prevalence of insomnia in OSA patients), the diagnostic tools and (severity) criteria for each disorder (e.g., requiring an AHI of five vs. 10 to diagnose OSA, or requiring formal diagnostic criteria for insomnia vs. self-reported symptoms via a brief questionnaire, etc.), and the population of interest (e.g., sleep clinic samples, general population, military personnel, etc.) [40]. Between 2013 and 2018, a number of large cluster analyses of OSA samples also identified that 32%–54% of OSA patients indicate symptoms of ‘disturbed sleep’, characterized by nocturnal insomnia symptoms, more frequent use of sedative-hypnotic medications, and lower use of CPAP therapy [26,27,41,42]. Most recently, Zhang and colleagues conducted a systematic review and meta-analysis including 37 studies investigating the co-morbidity of insomnia and OSA, and reported that 35% of insomnia patients have an AHI of ≥5, and 29% have an AHI of ≥15, while 38% of OSA patients meet insomnia criteria [43]. Interestingly, this meta-analysis excluded studies investigating veterans and samples comprised of trauma patients, in which even higher COMISA prevalence rates have been reported [44,45].

### 1.4. Consequences of COMISA

Importantly, COMISA patients experience greater impairments to daytime functioning and quality of life, compared to those with either insomnia, or OSA alone [40]. For example, Krakow and colleagues were among the first to report that compared to patients with OSA alone, COMISA patients expressed greater emotional and cognitive impairments, including irritability, reduced concentration, depressive symptoms, and anxiety [38]. In 2017, we reviewed a large number of studies which indicated an additive and substantial impairment to sleep, daytime functioning, depressive and psychiatric symptoms, and quality of life among COMISA patients [40]. Since 2017, a number of other studies have confirmed associations between COMISA and increased depressive and anxiety symptoms, daytime sleepiness, reduced quality of life, neurocognitive performance, and sleep quality compared to patients with either insomnia or OSA alone [46,47,48,49,50,51,52]. Hence, a large body of evidence has left little doubt that COMISA is a common disorder, which is associated with substantial impairments to nocturnal sleep, daytime functioning, and quality of life.

### 1.5. Refining the Measurement of COMISA

The presence and severity of insomnia has been defined according to a large range of measures and methods including structured and semi-structured interviews, self-report questionnaires, sleep diaries, and objective sleep recordings [53,54,55,56,57]. However, there has previously been a lack of research attention directed toward the validity of existing insomnia measures in the presence of OSA [58]. As insomnia and OSA share multiple overlapping symptoms, the diagnosis and measurement of each disorder in the presence of the other represents a complex task for researchers and clinicians [39,40,59].

Insomnia is diagnosed according to the frequency, severity, and chronicity of nocturnal sleep complaints and daytime impairments [1]. However, OSA commonly results in similar symptoms, including frequent post-apneic nocturnal awakenings, perceptions of nonrestorative sleep, daytime sleepiness and fatigue, and reduced quality of life [39,60]. It is possible that these shared symptoms result in an artefactual increase in the prevalence of COMISA, and complicate the assessment of changes in insomnia symptoms before and after different treatment approaches. For example, questionnaire measures of insomnia commonly include both nocturnal and daytime symptoms [54,55,61]. However, it is possible that many OSA patients may indicate elevated daytime impairments on these questionnaire measures in the absence of nocturnal insomnia complaints, resulting in higher overall ‘insomnia’ scores and a misclassification of COMISA. The same overlapping symptoms may also complicate the measurement of insomnia symptoms following different treatment approaches in COMISA. For example, COMISA patients treated with CPAP therapy may report a reduction of daytime impairments, which may translate to an overall reduction of ‘insomnia severity’ questionnaire scores in the absence of improved nocturnal symptoms.

To investigate the measurement of insomnia symptoms in the presence of OSA, Wallace and Wohlgemuth [62] recently examined profiles and predictors of insomnia severity index (ISI) questionnaire responses among 630 veterans with OSA. The ISI [54] is a brief, and valuable questionnaire measure of insomnia presence and severity, which has been utilized in several hundred insomnia treatment studies, including COMISA research [52,63,64,65]. The ISI includes seven self-report items, including three nocturnal items (difficulties falling asleep, staying sleep, and waking up too early), and four daytime items (satisfaction with sleep, daytime functioning interference, quality of life impairment, and worry/distress). These items are traditionally summed for a total score ranging from 0 to 28, with higher scores indicating more severe insomnia, and a score of ≥15 indicating clinically significant insomnia symptoms. Wallace and Wohlgemuth performed a latent profile analysis which identified that 30% of their OSA sample reported moderate insomnia, and an additional 44% reported severe insomnia. Importantly, these ‘moderate’ and ‘severe’ COMISA patients reported elevated nocturnal and daytime ISI symptoms. Wallace and Wohlgemuth propose that when administered to OSA patients, the ISI should be scored according to a ‘nocturnal’ sub-score (comprising the first three items, ranging from 0 to 12), and a ‘daytime’ sub-score (comprising the subsequent four items, ranging from 0 to 16). This ‘nocturnal’ score has also recently been investigated in other insomnia research [66,67], and has previously been utilized in several COMISA treatment studies [52,63,64].

It will be important for future research to validate different scoring criteria and cut-offs of the ISI and other insomnia questionnaire measures against psychologist-diagnostic criteria in the presence of OSA, to further refine the diagnosis and measurement of COMISA. Furthermore, future treatment research in COMISA samples should aim to include a range of outcomes measures which assess unique, and shared symptoms of each disorder.

## 2. Treatment of COMISA

### 2.1. Traditional Treatment Approaches

CPAP therapy is the most effective treatment for moderate and severe OSA [15,21]. Barthlen and colleagues were among the first to describe the association of insomnia symptoms and reduced CPAP adherence among two patients with severe OSA and insomnia symptoms [68]. Since this time, a large number of case studies [69], pilot studies [70,71], chart-reviews [65,72], treatment trials [44,49,50], and cluster analyses [41,42] have examined the effect of co-morbid insomnia symptoms on reduced CPAP outcomes in OSA patients. Although some studies report no association [63,73,74], the majority of research suggests that the presence of insomnia symptoms reduces CPAP acceptance and use. Understandably, patients who spend long periods awake wearing pressurized CPAP masks throughout the night, are more likely to experience CPAP-related side effects, and will be more likely to remove the CPAP equipment during the night, or reject CPAP therapy entirely.

This finding has led several groups to suggest that COMISA patients should be referred for insomnia treatment before commencing CPAP therapy. CBTi is the recommended ‘first line’ treatment for insomnia, and appears to be effective in the presence of co-morbid OSA [8,9,52]. Although several early pilot studies and a randomized controlled trial (RCT) reported mixed findings [69,75,76,77], more recent quasi-experimental and RCT data support the effectiveness of CBTi in COMISA patients [52,64,78,79]. For example, we previously reported a chart review of 455 insomnia patients treated with CBTi at an out-patient hospital insomnia treatment service, and found that there were no significant differences in insomnia improvements during treatment between the 141 patients with co-morbid OSA, and the 314 patients with insomnia alone [52]. Furthermore, there was no association between OSA severity (AHI) and changes in nocturnal insomnia symptoms during treatment, indicating that those without OSA and those with mild, moderate, and severe OSA experienced similar benefit from CBTi. Fung and colleagues, also recently compared the effectiveness of a five session CBTi intervention with a sleep-education control, among 134 adult veterans with insomnia alone (*n* = 39), and co-morbid insomnia and mild OSA (*n* = 95), and also found no differences in insomnia improvements during CBTi between patients with insomnia alone, and COMISA patients [78]. Finally, our recent RCT data from 145 COMISA patients with moderate and severe OSA, indicated that CBTi leads to greater improvement in ISI scores, diary-measured sleep parameters, and dysfunctional beliefs and attitudes about sleep by post-treatment, compared to a no-treatment control group [64].

Given the effect of co-morbid insomnia symptoms on reduced acceptance and use of CPAP therapy [40], it is also important to consider the effectiveness of non-CPAP therapies in the COMISA population. A small number of studies have investigated the effect of oral appliance devices, upper airway surgery, and nasal dilator strip therapy in the treatment of COMISA patients [75,80]. For example, Guilleminault and colleagues [77] reported that upper airway surgery significantly improved sleep parameters, sleep architecture, AHI, and daytime functioning in 30 patients with co-morbid insomnia and mild OSA. Alternatively, Krakow and colleagues reported that nasal dilator strip therapy improves insomnia symptoms and perceived sleep quality in patients with subjective symptoms indicative of sleep maintenance insomnia and OSA [81,82]. Future research should continue to examine the efficacy of these and other non-CPAP therapies among COMISA patients unable to tolerate CPAP therapy (e.g., oral appliance devices, positional devices, etc.).

### 2.2. Combined Treatments for COMISA

Krakow and colleagues were among the first to propose that COMISA patients have greater difficulties adapting to CPAP, and should be referred for CBTi before commencing CPAP therapy [38]. Two early studies provided preliminary support for the combination of CBTi and CPAP therapy in COMISA patients [76,83]. In 2001, Melendrez and colleagues reported a study examining the effect of CBTi followed by CPAP therapy in seven female crime victims with PTSD and COMISA [76]. They found that the patients experienced a significant six points ISI reduction following CBTi, and an additional six points ISI reduction following three subsequent months of CPAP therapy. Alternatively, Wickwire and colleagues reported a case study in which a middle-aged male with chronic insomnia and OSA was treated with a nine session CBTi program, which resulted in a small increase in CPAP use [83]. 

Until very recently, there have been very few trials examining the combination of CBTi and CPAP therapy in COMISA patients [39,40]. Table 1 displays an overview of recent RCTs investigating the combination of CBTi and CPAP therapy in COMISA. Bjorvatn and colleagues recently reported an RCT in which 134 COMISA patients were randomized to receive either a self-help CBTi book, or sleep hygiene information while commencing CPAP therapy [84]. The same research group previously found that the CBTi book resulted in significantly greater improvements in insomnia symptoms compared to sleep hygiene information material, among patients with insomnia alone [85]. In their recent COMISA study, although both the CBTi book and sleep hygiene control groups reported significant improvement of insomnia symptoms from pre- to post-treatment (change in ISI, and Bergen Insomnia Scale [86]), there was no difference in improvements between groups [84]. Similarly, there were also no differences in objective CPAP adherence over the first 3 months of treatment between groups (2.54 hours, versus 2.55 hours in the sleep hygiene and CBTi book groups, respectively). This lack of differences in insomnia and CPAP outcomes may be due to this sample reflecting patients primarily recruited for management of OSA, who were potentially less motivated to read and adhere to the instructions of the CBTi book. Indeed, 22% of the COMISA patients reported that they did not read the CBTi material, compared to only 5% of insomnia patients investigated in the previous RCT [84,85]. As the CBTi book and CPAP were commenced concurrently, it is also possible that patients derived sufficient benefit from CPAP alone, and did not perceive a need to utilize the CBTi intervention.

Alessi and colleagues recently reported the preliminary results of an RCT [79,87] comparing the effect of a five session combined CBTi and behavioral CPAP adherence program delivered by trained ‘sleep coaches’, versus a sleep education control program, on insomnia improvement and CPAP use by 6 month follow-up. The CBTi/adherence intervention was delivered concurrently with CPAP therapy (the first session was administered prior to commencing CPAP therapy, and the subsequent sessions were administered after commencing CPAP therapy). They recruited 125 adult veterans (96% male) with ICSD-3 insomnia, and an AHI of at least 15 (indicating moderate and severe OSA, however average AHI for the group was in the severe range; Mean AHI = 35). Compared to sleep education control, the CBTi group showed a greater improvement of diary- and actigraphy-measured sleep parameters, greater ISI reduction, and 78 and 48 min greater adherence to CPAP therapy at the 3 and 6 month follow-up, respectively. 

Ong and colleagues also recently reported the preliminary results of an RCT [88,89] comparing the effects of three treatment approaches for COMISA on insomnia symptoms and CPAP adherence over the first 3 months. Intervention-arms included administering CBTi before commencing CPAP therapy, administering CBTi concurrently with CPAP therapy, and treating patients with CPAP therapy alone. Patients included 121 adults with ICSD-2 insomnia, and an AHI of at least five (although average AHI for the whole sample was 24, indicating moderate-to-severe OSA). There was a significant group by time interaction on ISI scores (*p* < 0.001), indicating that the two groups of patients receiving both CBTi and CPAP therapy showed a greater reduction of global insomnia severity during treatment, compared to patients receiving CPAP alone. Alternatively, there was no difference in ISI improvement between groups receiving initial, versus concurrent CBTi. There was no difference in CPAP adherence between any of the three groups by 3 months follow-up. Full results of this study are yet to emerge.

Finally, we recently reported an RCT comparing the effect of a four sessions individual/small group CBTi program, versus a no-treatment control group, on improvements in insomnia symptoms and subsequent acceptance and long-term objective use of CPAP therapy [64]. The manualized CBTi program was delivered by psychologists, and included sleep hygiene education, sleep restriction therapy, cognitive therapy, sleep misperception feedback, and relapse prevention. We recruited 145 patients with ICSD-3 insomnia, and an AHI of at least 15 (although average AHI for the sample was in the severe range; *M* = 35) from a Hospital Sleep Clinic, and an online advertising recruitment arm. Compared to the control group, we found that the CBTi group showed significantly greater improvement of sleep-diary parameters, the ISI, and dysfunctional sleep-related beliefs from pre- to post-CBTi, and subsequently showed greater initial acceptance of CPAP, and 61 min greater use by 6 months follow-up [64]. Like Alessi and colleagues [79], and Ong and colleagues [88], we also found that COMISA patients receiving both CBTi and CPAP therapy reported significantly greater improvement of global insomnia symptoms by 6 months follow-up.

### 2.3. Summary of Recent COMISA Randomized Controlled Trials

Together, these recent larger RCTs provide tentative support for the effect of therapist-administered CBTi in improving insomnia symptoms and increasing subsequent use of CPAP therapy in COMISA patients. Firstly, it appears that CBTi delivered by trained therapists may be more effective than self-administered (bibliotherapy) CBTi materials for COMISA patients. Although Bjorvatn previously found that a self-administered CBTi book resulted in significantly greater insomnia improvements compared to a sleep hygiene control group in patients with insomnia alone [85], their more recent study indicated that COMISA patients were less likely to read the CBTi material, and showed no differences in insomnia improvements or CPAP use compared to the control group [84]. Alternatively, the recent RCTs [64,79,88] and previous studies [52,78] investigating therapist-delivered CBTi, indicate that when delivered in a face-to-face setting, CBTi is an effective insomnia treatment in the presence of co-morbid OSA. An additional study is currently investigating the effect of self-administered online CBTi in the treatment of COMISA [90].

Secondly, the avenue of patient referral and recruitment may impact the effectiveness of different treatment approaches. As discussed by Bjorvatn [84], patients presenting with a ‘chief complaint’ of OSA may show reduced motivation to adhere to CBTi instructions, and will experience little additional benefit from initial treatment with CBTi. Alternatively, patients presenting for the diagnosis/treatment of insomnia may be more likely to engage with CBTi, and therefore experience a greater effect of CBTi on improved sleep and CPAP outcomes. Differences in sample populations, recruitment, presenting symptoms and motivation for different treatments may partially explain the differences between these RCTs. For example, while Bjorvatn [84] primarily recruited patients referred for suspicion of OSA and found no effect of a self-administered CBTi book, Ong and colleagues recruited patients from a combination of community advertisements, word of mouth, referrals from other physicians and healthcare providers, and a pool of previous research participants, and found that CBTi effectively improved insomnia symptoms [89]. Alternatively, the participants in our study [64] were recruited from both sleep clinic populations (comprised of patients seeking a diagnosis and treatment for OSA), and an online recruitment arm (comprised of patients self-referred with symptoms indicative of insomnia and/or OSA), and showed a positive response to CBTi. The full results and recruitment pathways of Alessi and colleagues’ study are yet to be reported [79]. It will be important for future research to investigate the effectiveness of different combined and singular treatment approaches, between patients presenting with a ‘chief complaint’ of either insomnia, OSA, or both disorders.

Regarding the sequential, versus concurrent delivery of CBTi and CPAP therapy, more data are needed to confirm the results and differences between these current trials, and guide clinical recommendations. While we demonstrated that initial treatment with CBTi was effective in improving initial acceptance and use of CPAP therapy [64], Alessi and colleagues [79] reported that concurrent delivery of CBTi increased CPAP use, while Ong and colleagues [88] reported no difference between initial or concurrent CBTi administration on CPAP outcomes, and Bjorvatn and colleagues [84] reported no effect of the concurrent administration of a CBTi book on insomnia or CPAP outcomes. Hence, there are substantial differences between these recent RCTs regarding the most effective sequence of treatments for COMISA. Given that co-morbid insomnia symptoms reduce acceptance of CPAP therapy, and that initial CPAP acceptance and use predicts future CPAP use [44,91], our recommendation is that CBTi should be initiated before commencing CPAP therapy to improve insomnia symptoms, and provide the greatest opportunity for a positive initial experience with CPAP therapy to encourage and maintain greater long-term adherence. However, the severity of patients’ insomnia and OSA, and patient preferences for CBTi versus CPAP therapy as the initial treatment should also guide decisions regarding the use and timing of CBTi and CPAP therapy [92]. More research is also needed to determine whether any baseline symptoms or profiles predict the success of different sequences of CBTi and CPAP therapy in COMISA [40].

Alessi and colleagues’ study [79,87], and our study [64] also included between-group CPAP data at both 3 and 6 month follow-up. Interestingly, while Alessi and colleagues observed a small decrease in CPAP use between the CBTi and control group between 3 month (CBTi group showed 78 min greater adherence) and 6 month follow-up (CBTi group showed 48 min greater CPAP adherence), we observed a stable maintenance of improved CPAP use in the CBTi group between 3 months (57 min greater adherence) and 6 months (64 min greater adherence). This difference in the maintenance of average CPAP use between CBTi and control groups, in these two studies may have resulted from the different control conditions (i.e., Alessi utilized a sleep education control, while we used a no-treatment control), or differences between study samples (e.g., Alessi’s sample primarily included male veterans, while we recruited non-veteran patients recruited through a Hospital Sleep Clinic). Despite these small differences in between-group CPAP use, both the CBTi and control groups in each study appeared to show a pattern of gradually declining CPAP use over time.

Finally, while the CBTi interventions administered by Ong [88,89], Bjorvatn [84], and our own study [64] included multi-faceted CBTi approaches to target insomnia, Alessi and colleagues’ intervention included a combination of CBTi components and CPAP-adherence strategies to simultaneously improve insomnia and encourage greater CPAP outcomes [79,87]. We chose to administer isolated treatments for insomnia, and OSA to investigate the relative contribution of each intervention to symptoms of COMISA [64]. However, given the success of previous motivational interviewing [93] and educational strategies [94] in improving CPAP acceptance and use, this combined strategy may prove to be the most effective for COMISA patients treated in clinical settings. Furthermore, as sleep clinics are encouraged to offer educational support for OSA patients commencing CPAP therapy [15], it may be possible to include CBTi interventions within these existing education platforms, to simultaneously target both insomnia symptoms and improved CPAP use [79].

## 3. Bi-Directional Relationships in COMISA

The high co-morbidity of insomnia and OSA are suggestive of underlying mechanistic bi-directional relationships, whereby symptoms of each disorder may pre-dispose patients to the development, or exacerbate the severity of the other [95,96]. For example, sleep loss may exacerbate manifestations of OSA. Several pilot-studies have reported that a full night of sleep deprivation reduces upper-airway muscle tone in normal sleepers [97,98,99], and results in an increased AHI and reduced minimum oxygen saturation in patients with suspected and mild OSA [100,101,102,103]. Furthermore, some evidence suggests that consecutive nights of partial sleep deprivation increase the frequency of respiratory events in patients with mild and moderate OSA [104]. Hence, it is possible that multiple consecutive or intermittent nights of partial sleep loss experienced by patients with chronic insomnia may contribute to the development or exacerbation of OSA.

Alternatively, insomnia disorder may be associated with a reduced respiratory arousal threshold, which may predispose patients to prematurely awaken to respiratory events [16,105]. Indeed, insomnia has been conceptualized as a disorder of chronic ‘hyperarousal’, including elevated cognitive (ruminations, anxiety, etc.) and physiological arousal (increased heart rate, sympathetic nervous system activity, etc.), which may include a reduced respiratory arousal threshold [3,106]. A greater frequency of post-apneic arousals and sleep onset events may increase time in ‘transitional’ light sleep and delay a patient’s progression into deep sleep which is associated with a reduced AHI [107]. Interestingly, Janssen and colleagues recently reported case study data from a 75 year old male in which a CPAP adherence data indicated an increased residual AHI during acute episodes of stress-induced insomnia symptoms [108]. Given the possible role of insomnia symptoms in the development and exacerbation of OSA, it will be important for future research to examine the impact of treating the insomnia, on changes in the onset, frequency, and duration of respiratory events in patients with COMISA [109,110].

Alternatively, OSA may contribute to the development or exacerbation of insomnia. OSA is associated with frequent post-apneic arousals and surges in sympathetic activity throughout the night, which may lead to full awakenings, and insomnia complaints. Mercer and colleagues [111] previously demonstrated that upon awakening throughout the night, insomnia patients commonly misperceive prior sleep as wakefulness. It is possible that among COMISA patients, post-apneic awakenings are also associated with sleep-state misperceptions, culminating in perceptions of prolonged time to fall asleep initially or prolonged awakenings during the night and thus an insomnia complaint. Indeed, previous research has reported a reduction in sleep maintenance insomnia complaints among COMISA patients who are able to tolerate CPAP therapy [63,112]. Although insomnia symptoms may be initially precipitated by post-apneic arousals in many patients, insomnia disorder quickly develops functional independence of the initial precipitating factors, and demands targeted treatment approaches (see discussion of ‘secondary’ versus ‘co-morbid’ insomnia, above) [4,33]. 

Although several research groups have discussed the potential bi-directional relationships between COMISA, there has been a lack of research attention in this important area [39,40,95,108]. It will be possible to use data from current RCTs to examine the independent effects of CBTi and CPAP therapy on changes in the ‘downstream’ severity and manifestations of each disorder. Furthermore, this may provide a platform to investigate predictors of which patients show the greatest response to insomnia and OSA treatments in isolation.

## 4. Recommendations for Clinicians

- The majority of sleep clinics around the world currently specialize in the diagnosis and treatment of OSA whist neglecting the measurement and treatment of insomnia. However, 30%–50% of OSA patients report co-morbid insomnia symptoms, which reduce acceptance and use of CPAP therapy.
- Co-morbid insomnia symptoms commonly reduce CPAP use and contribute to higher impairment of daytime functioning and quality of life. Therefore, the insomnia symptoms demand targeted diagnostic and treatment considerations, and should not be assumed to be a ‘secondary’ manifestation of the OSA.
- Clinicians should administer sleep diaries [53], or the insomnia severity index [54] which can be scored with adjusted COMISA cut-offs [62], to screen for insomnia symptoms in patients with suspected OSA.
- COMISA patients should be treated with both CBTi and CPAP therapy to improve insomnia symptoms, and increase CPAP acceptance and use.
- CBTi should be delivered by psychologists, or trained therapists, who can also provide motivational CPAP support.

## 5. Future Research Directions

- The diagnosis of co-morbid insomnia and OSA represents a complex task due to shared diagnostic symptoms. It is important to validate and refine insomnia measures in the presence of OSA.
- Investigate baseline symptoms and profiles which predict successful responses to different treatment combinations and sequences in COMISA.
- Investigate bi-directional relationships between COMISA, by examining 1) the effect of CBTi on manifestations and severity of OSA (e.g., AHI [109]), and 2) examine the effect of CPAP therapy on manifestations and severity of insomnia symptoms (e.g., sleep parameters, sleep misperceptions, ISI, etc.).
- Determine the most effective CBTi components and combinations to treat insomnia and improve CPAP adherence (for example, using isolated CBTi components such as bedtime restriction therapy to increase sleep efficiency before commencing CPAP therapy).
- Continue examining the efficacy of non-CPAP therapies in the presence of co-morbid insomnia symptoms, for patients who reject CPAP therapy.

## 6. Conclusions

Although COMISA was first identified by Guilleminault and colleagues in 1973, there was a lack of research attention until the publication of two papers in 1999 and 2001 indicating a 30%–50% co-morbid prevalence rate. Subsequent research indicated that COMISA patients experience greater impairment of sleep, daytime functioning, and quality of life, compared to patients with either insomnia, or OSA alone. However, despite these additive impairments, COMISA patients also show worse acceptance and use of CPAP therapy, compared to patients with OSA alone. A number of recent RCTs have provided evidence that CBTi is an effective insomnia treatment in the presence of co-morbid OSA. Furthermore, COMISA patients treated with CBTi show increased initial acceptance and long-term use of CPAP therapy, compared to treatment with CPAP alone. It is recommended that clinicians utilize simple instruments to detect insomnia symptoms in OSA patients, and refer identified COMISA patients for combined CBTi and CPAP therapy. It will be important for future research to examine mechanistic bi-directional relationships between COMISA, and continue to refine and tailor different treatment combinations and sequences.

## Figures and Tables

**Table 1:** Recent randomized controlled trials investigating the combination of CBTi and CPAP therapy for COMISA patients.

Study	*n*	Diagnostic Criteria	CBTi Intervention	Control	CPAP Follow-up	Insomnia Outcome	CPAP Use
Alessi et al., 2018 [79,87]	125	ICSD-3, AHI ≥ 15	Five session CBTi and behavioral CPAP adherence program, delivered concurrently with CPAP	Sleep Education Program	Objective CPAP data at 6 months.	CBTi group showed greater improvement during treatment.	CBTi group showed 78, and 48 min greater CPAP use, at the 3 and 6 month follow-ups, respectively.
Bjorvatn et al., 2018 [84]	134	DSM-5, ICSD-3, AHI ≥ 5	Previously validated self-help CBTi book, delivered concurrently with CPAP	Sleep hygiene advice	Objective CPAP data at 3 months.	No difference in improvement of ISI or Bergen Insomnia Scale between groups.	No significant difference between groups. Mean difference = 1 minute.
Ong et al., 2019 [88,89]	121	ICSD-2, AHI ≥ 5	Four session CBTi program, delivered before vs. concurrently with CPAP	No treatment, monitoring	Objective CPAP data at 3 months	CBTi groups reported greater ISI improvement during treatment.	No significant difference between CBTi and CPAP-only groups. CBTi before CPAP (*M* use = 148 min, *SD* = 137) CBTi with CPAP (*M* use = 152 min, *SD* = 155) CPAP only (*M* use = 181 min, *SD* = 155).
Sweetman et al., 2019 [64]	145	ICSD-3, AHI ≥ 15	Four session CBTi program delivered before CPAP	No treatment	Objective CPAP data at 6 months.	CBTi group reported greater improvement of the ISI, sleep diary parameters, and dysfunctional beliefs about sleep during treatment.	CBTi group showed 61 min greater CPAP use over the first 6 months. CBTi before CPAP (*M* use = 265, *SD* = 166) CPAP only (*M* use = 204, *SD* = 153).

AHI = Apnea/hypopnea index, CBTi = cognitive and behavioral therapy for insomnia, CPAP = continuous positive airway pressure therapy, DSM-5 = Diagnostic and statistical manual of mental disorders 5th ed, ICSD = International classification of sleep disorders, ISI = insomnia severity index, SD = standard deviation.

## Abbreviations

AHI	Apnea/hypopnea index
CBTi	Cognitive and behavioral therapy for insomnia
COMISA	Co-morbid insomnia and sleep apnea
CPAP	Continuous positive airway pressure
ISI	Insomnia severity index
OSA	Obstructive sleep apnea
PSG	Polysomnography
RCT	Randomized controlled trial
